# Emergency endovascular treatment of stroke due to cervical artery dissection – impact of periprocedural GP IIb/IIIa inhibitor use on clinical outcome

**DOI:** 10.1186/s42155-025-00564-9

**Published:** 2025-05-22

**Authors:** Abira Sornalingam, Susanne Wegener, Miranda Stattmann, Jil Baumann, Patrick Thurner, Jawid Madjidyar, Hakim Shakir Husain, Miklos Krepuska, Christoph Globas, Andreas R. Luft, Zsolt Kulcsar, Tilman Schubert

**Affiliations:** 1 Department of Neuroradiology, University Hospital Zurich, Frauenklinikstrasse 10, Zurich, 8091 Switzerland; 2 Department of Neurology, University Hospital Zurich, Zurich, Switzerland; 3 Cereneo Center for Neurology and Rehabilitation, Vitznau, Switzerland; 4 Baby Memorial Hospital, Calicut, Kerala India; 5 Parco Institute of Medical Sciences, Vatakara, Kerala India; 6 Neo Hospital, Noida, Uttar Pradesh India

**Keywords:** Cervical artery dissection, Ischemic stroke, Endovascular thrombectomy, GP IIb/IIIa inhibitor

## Abstract

**Background:**

Endovascular therapy (EVT) for large intracranial vessel occlusion or symptomatic hypoperfusion due to cervical artery dissection (CeAD) became the standard therapy in recent years. Stenting is frequently required to secure the dissection with subsequent need for GP IIb/IIIa inhibitors. However, a potential concern of antithrombotic therapy in acute stroke is the increased risk of intracerebral hemorrhage.

The aim of the study was to assess the impact of the administration of a GP IIb/IIIa inhibitor imaging during endovascular treatment for acute ischemic stroke caused by CeAD on 90-day clinical outcome and intracranial hemorrhage.

**Methods:**

This single-center retrospective cohort study enrolled CeAD patients with internal carotid artery (ICA) dissections treated with EVT from January 2015 to August 2022. We analysed the impact of different variables including postinterventional hemorrhage, revascularization success and the use of GP IIb/IIIa Inhibitors (eptifibatide) on 90-day favorable clinical outcome (mRS 0–2). NIHSS Scores were evaluated at different time points in relation to the 90-day clinical outcomes.

**Results:**

Forty-nine patients were included in the study. Thrombectomy was performed in all patients. In 33 patients, stenting was performed in addition to thrombectomy. 20 patients (40.8%) received eptifibatide periinterventionally. 31 out of 49 patients (63.3%) had a favorable 90-day clinical outcome (mRS 0–2). Five patients showed radiologically significant hemorrhage. The rate of successful reperfusion (TICI 2b-3) in the favorable 90-day outcome group was significantly higher than in the unfavorable 90-day outcome group.

**Conclusions:**

In this study, the use of a GP IIb/IIIa inhibitor use during EVT for stroke caused by CeAD did not affect 90-day clinical outcome nor the incidence of intracranial hemorrhage. Successful reperfusion significantly correlated with favorable clinical outcome.

## Introduction

Cervical artery dissection (CeAD) is a rare cause of acute ischemic stroke. It occurs in less than 2.5% of all stroke patients. However, this etiology is of great importance among patients under the age of 45, where it is responsible for 10% to 25% of all ischemic stroke cases [1].

CeAD is the result of a subintimal split of the internal carotid or vertebral arterial wall which leads to intramural hematoma formation. The formation of an intramural hematoma, known as a false lumen, can lead to thrombosis, stenosis, and intracranial embolic occlusions [1]. Nevertheless, the pathogenesis of a CeAD is not evidently explained [2]. The majority of CeAD appears spontaneously with or without an underlying predisposition. Further frequent causes include minor head and neck trauma. Genetic risk factors include fibromuscular dysplasia (FMD), alpha-1-antitrypsin deficiency, hyperhomocysteinemia or connective tissue diseases like Ehlers-Danlos syndrome type IV and Marfan’s syndrome [3–5].

Due to the variety of clinical manifestations of CeAD, the clinical features may appear to be the same as those characteristics of a stroke. The most common local symptoms are headache, ipsilateral head, neck and face pain, Horner syndrome and dizziness. Moreover, approximately two-thirds of all CeAD patients suffer from an ischemic stroke or a transient ischemic attack [6, 7]. Approximately 5% of CeAD patients are asymptomatic [4].

The treatment options for CeAD depend on the clinical manifestations and focus on reducing the rate of ischemic stroke recurrence. Medical treatment is based on antithrombotic therapy with anticoagulants or antiplatelet drugs [8], whereby the clinical trials (CADISS, TREAT-CAD) show no difference in efficacy between that drugs [9, 10].

Efficacy concerns regarding intravenous thrombolysis (IVT) in strokes related to CeAD exist [11], however, as this specific question has not been answered by randomized controlled trials, IVT is widely used in eligible CeAD patients [12, 13].

Endovascular therapy (EVT) is only required in cases of symptomatic cervical artery stenosis or occlusion, such as accompanied by neurological deficits or ischemic events, including stroke or TIA, or intracranial large vessel occlusion [14].

Procedures in CeAD are generally more complex than limited intracranial thrombectomies, due to the necessity to cross and treat the cervical lesion together with the intracranial occlusion. In addition, stents are frequently needed to treat the cervical lesion, which requires double antiplatelet therapy and may increase the risk of hemorrhage in case of stroke.

Multiple observational case studies have been conducted to demonstrate the safety and effectiveness of endovascular therapy in acute ischemic strokes due to CeAD in recent years, which show favorable outcomes, feasibility and efficacy [15–21]. However, due to the limited data available considering CeAD-related stroke, the outcomes of EVT in CeAD may still be understudied [22–25].

Glycoprotein GP IIb/IIIa inhibitors have shown promise as short-acting, selective, and reversible antiplatelet agents in the treatment of acute ischemic stroke. Initially designed for treatment of myocardial infarction [26], GP IIb/IIIa inhibitors are nowadays widely used to prevent thromboembolic complications after emergency stenting for ischemic stroke treatment. Safety studies, including the CLEAR trial in 2008 and a meta-analysis, have demonstrated that eptifibatide, either isolated or in combination with rt-PA, does not significantly increase the risk of symptomatic intracranial hemorrhage [27–29].

In this study, we evaluated the outcome of EVT for CeAD with and without epftifibatide andmistration. We assessed the clinical outcome with special regard to the safety and efficacy of GP IIa/IIIb antagonist administration (eptifibatide) in terms of 90-day clinical outcome and postinterventional radiologically significant intracranial hemorrhage.

## Methods

In this single-center cohort study, we retrospectively evaluated all admitted patients with CeAD-related (internal carotid artery) ischemic stroke, which underwent EVT at our institution between 1. January 2015 and 31. August 2022. General informed consent was obtained from all subjects, the study was approved by the cantonal ethics committee Nr. 2018–01212.

In the current study, we searched our internal stroke database for CeAD-related stroke patients treated with EVT. Consecutive patients with the internal carotid artery CeAD suffering an ischemic stroke were included.

We assessed the following clinical and radiological data: age and sex; standard stroke severity measured by the National Institutes of Health Stroke Scale (NIHSS); Modified Ranking Score (mRS) on admission; the pre-Alberta Stroke Program Early CT Score (ASPECTS) from radiological brain imaging, preoperative antithrombotic management, EVT technique, TICI-Score, post-operative occlusion within 24 h, intraoperative antithrombotic medication and periinterventional complications. In addition, the incidence of elongated styloid process, trauma and subpetrous carotid loops were recorded in order to investigate a potential association with ICA dissections.

### Endovascular therapy

Mechanical thrombectomy procedures were performed under general anesthesia (GA). An 8 F short sheath and a balloon guide catheter were used for access, whenever the vascular anatomy allowed for. Patients without intravenous thrombolysis received an initial bolus of intravenous heparin (2000–3000 IU). For revascularization, the distal-to-proximal concept was favorized wherever possible, aiming for the intracranial mechanical thrombectomy first and stent reconstruction of the cervical carotid artery second. For the procedure a balloon guide catheter (Flowgate 8 F, Stryker, Irvine, California, USA, or Bobby, Microvention, Aliso Viejo, CA, United States) was used as standard, and the thrombectomy was performed either with direct aspiration, stent retrieval or the combination of those.

After intracranial revascularization, stent reconstruction of the carotid lesions was considered, depending on the flow-relevance of the lesion. In case of stent placement, patients were given 300 mg of aspirin intravenously, regardlessly of IVT with rtPA.. Different types of stents were available to reconstruct the internal carotid artery (Casper, Onyx, Leo, Precise, Solitaire AB, Zilver Carotid). If single or dual layer stents were utilized was recorded.

A GPIIb/IIIa antagonist (Eptifibatide (Integriline, GSK, London, UK)) was given if there were signs of early (after 15 min) re-thrombosis after stent placement, with a weight-adapted 180 µg/kg bolus followed by 2,0 µg/kg/min maintenance dose) and was continued 6–24 h after the procedure. The subsequent decision on single or double oral antiplatelet therapy (SAPT or DAPT) was taken post procedurally based on the vessel patency, infarct size, and the presence of hemorrhage. Heparinized saline (1250 IE Heparin/1000 ml saline) was used to continuously flush catheters.

### Postinterventional follow-up

The follow-up data contained: postinterventional 24-h radiological brain imaging; mRS at discharge; 90-day mRS; NIHSS at discharge; 90-day NIHSS;. The cutoff for a favorable 90-day mRS was defined as 0–2 (functional independence in daily life). A TICI score of 2b-3 was defined as successful recanalization.

Postintervention intracranial hemorrhages after EVT were categorized by the Heidelberg Bleeding Classification based on 24-h follow-up brain imaging. (0 = no bleeding, 1 = hemorrhage transformation, 2/3 = significant/severe hemorrhage) [30]. Exclusion criteria were initial modified Ranking Score (mRS) greater than 3, loss of follow-up, procedural complication as perforation, non-carotid artery dissection related strokes and iatrogenic generated CeAD [31–33].

### Outcome variables

**Fig. 1:**
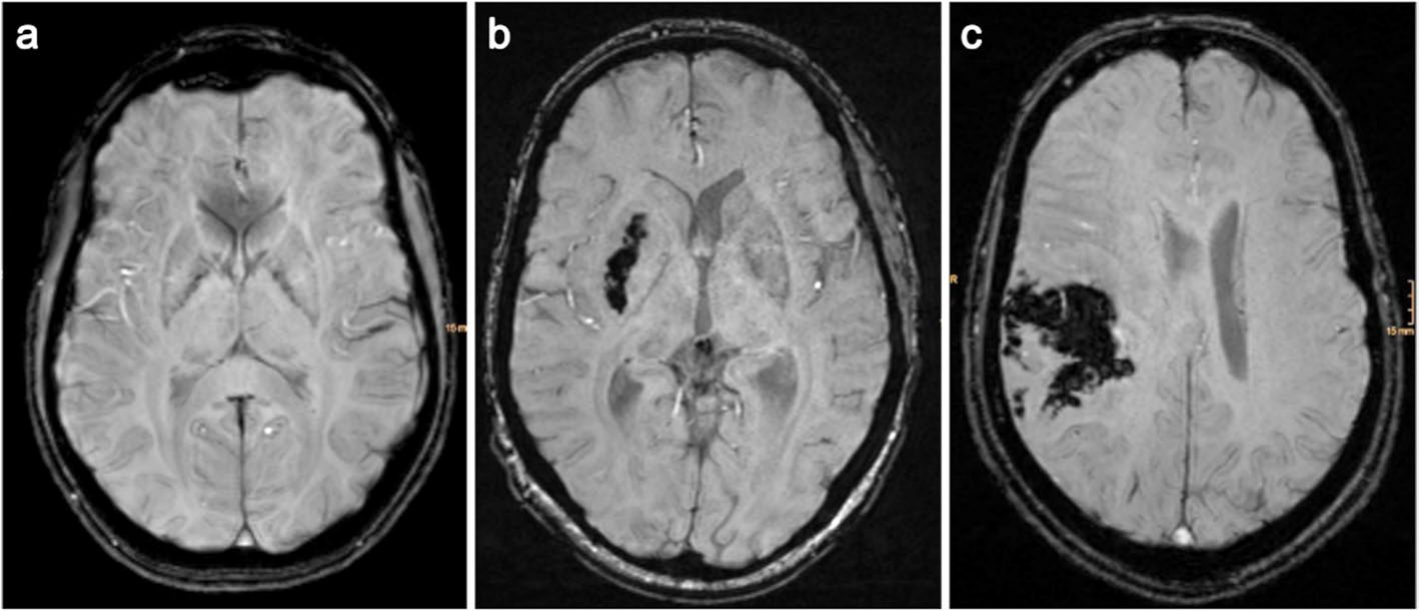
Representative examples of postinterventional 24-h follow-up susceptibility-weighted imaging (SWI) on MRI; hemorrhages classified by The Heidelberg Bleeding Classification: **a** No hemorrhagic transformation (Heidelberg Class 0). **b** Hemorrhagic transformation (Heidelberg Class 1). **c** Significant hemorrhage (Heidelberg Class 2)

To determine the impact of eptifibatide on outcome, a comparison analysis between patients who received periinterventional antithrombotic therapy with eptifibatide and those who did not, was performed. The outcomes of the study were three-fold, including (i) favorable 90-day modified Rankin Scale (mRS) outcome, (ii) radiological significant hemorrhage detected through radiological imaging within 24 h; Heidelberg Classification Score = 2/3 (Fig. 1) and (iii) successful revascularization TICI-Score 2b-3.

Furthermore, the analysis assessed the difference in NIHSS at admission, discharge, and the 90-day follow-up as well as the use of periprocedural heparin and aspirin between patients with favorable and unfavorable clinical outcomes.

### Statistical analysis

The statistical analyses for this study were conducted using The R for Statistical Computing software. Initially, descriptive statistics were generated to provide an overview of the demographics and clinical features of patients with CeAD who underwent EVT.

Pearson and Fisher’s exact chi-square tests were used to analyze categorical variables, while Wilcoxon-Mann–Whitney rank sum-tests were used for continuous variables such as NIHSS and Pre-ASPECT scores. Data variables were presented as percentages, mean with standard deviation, or median with interquartile range (IQR). A significance level of *p* < 0.05 was used, and the *p-*values were adjusted by Benjamini and Hochberg as a multiple testing correction to reduce the risk of Type I errors (false positives).

## Results

Among 154 patients diagnosed with CeAD, 65 patients underwent endovascular therapy. 49 subjects met the eligibility criteria for this study. Criteria for exclusion were missing informed consent (*n* = 3), loss of follow-up (*n* = 4), preinterventional modified Ranking Score (mRS) greater than 3 (*n* = 1), periprocedural vessel perforation with intracranial hemorrhage (*n* = 2), vertebral artery dissection (*n* = 4) and iatrogenic CeAD (*n* = 2), in total *n* = 12.

The median age was 61 years and 35 patients were male (71.4%). Among the 49 patients who suffered a stroke due to CeAD have an occlusion of the extracranial internal carotid artery (ICA), leading to extracranial—intracranial tandem occlusions that typically involved the middle cerebral artery (MCA) apart from one individual with CeAD without carotid occlusion and embolic M1-occlusion.

The median baseline NIHSS was 12/42 at hospital admission. On admission, the median pre-ASPECTS was 7.5/10 (summarized in Table 1).

**Table 1:** Clinical characteristics of all included patients with EVT in the study. Summary of patient characteristics including clinical- and imaging data as well as intra- and preprocedural antithrombotic and anticoagualtion medication (numbers are individual patient counts and percent in brackets, Inter Quartile Range in square brackets)

Variables	Overall
Total	49
Age (median [IQR])	61.00 [53.00, 68.00]
Sex = Male (%)	35 (71.4)
mRS on admission 0–2 (%)	12 (24.5)
mRS at discharge 0–2 (%)	19 (38.8)
mRS at 90-day 0–2 (%)	31 (63.3)
NIHSS on admission (median [IQR])	12.00 [6.00, 16.00]
NIHSS at discharge (median [IQR])	5.00 [1.00, 10.00]
NIHSS at 90-day (median [IQR])	2.00 [0.00, 5.00]
Pre-ASPECTS (median [IQR])	7.50 [6.75, 9.00]
Pre-event ASS intake	7 (14.3)
Pre-event anticoagulation intake	2 (4.1)
IVT rt-PA (Alteplase)	28 (57.1)
Eptifibatide administration	20 (40.8)
Elongated Processus styloideus (%)	15 (30.6)
Trauma (%)	49 (100.0)
Subpetrous Loop (%)	21 (42.9)
IAT stenting (%)	33 (67.3)
Dual layer of stents (%)	
No	25 (51.0)
Yes	8 (16.3)
Stents number (%)	
0	16 (32.7)
1	19 (38.8)
2	9 (18.4)
3	4 (8.2)
4	1 (2.0)
TICI (%)	
0	5 (10.2)
1	1 (2.0)
2b	11 (22.4)
2c	5 (10.2)
3	27 (55.1)
Post-OP reocclusion within 24 h (%)	5 (10.2)
ICA occlusion posttreatment (%)	9 (18.4)
Bleeding Classification Heidelberger (%)	
No bleeding (0)	29 (59.2)
Hemorrhagic transformation (1)	15 (30.6)
Radiologically significant bleeding (2/3)	5 (10.2)

### Endovascular therapy

Mechanical thrombectomy was performed using either a stent retriever with or without additional distal aspiration in 47 patients. Aspiration alone was used in 2 cases. Endovascular thrombectomy was performed in 16 subjects without stenting of the dissection. In 33 patients, stenting was performed in addition to thrombectomy, one patient was treated solely with stenting of the cervical lesion without intracranial thrombectomy. In case of dissections extending from the cervical to the intracranial ICA, the vessel was reconstructed using overlapping stents. 19 patients were treated with a single stent. Two stents were used in 9 patients while three stents were needed in 4 patients. Only one patient required four stents (Table 1). The stents used were Casper ((Microvention, Aliso Viejo, CA) *n* = 13, ranging from 5 × 30–8 × 40 mm), Onyx ((Medtronic, Dublin, Ireland) *n* = 1, 4.5 × 15 mm), Leo ((Balt, Montmorency, France) *n* = 8, 5 × 20–5.5 × 50 mm), Precise ((Cordis, Miami Lakes, FL) *n* = 14, 5 × 40–9 × 40 mm), Solitaire AB ((Medtronic, Dublin, Ireland) *n* = 13, 5 × 20–6 × 30 mm), Zilver Carotid ((Cook Medical, Bloomingtom, IN) *n* = 4, 6 × 30–7 × 80 mm). 13 dual layer stents were utilized.

Successful recanalization (TICI 2b-3) was achieved in 43 of 49 patients (87.7%). Unsuccessful revascularization (TICI 0–1) occurred in 6 cases (12.2%).

Twenty patients (40.8%) received eptifibatide during the procedure due to thrombotic phenomena.

Twenty-eight patients (57.1%) received intravenous rt-PA prior to EVT within 4.5 h from symptoms onset (Table 1). Nine patients (19.4%) out of 49 were on antiplatelet drugs (*n* = 7) or anticoagulants (*n* = 2) prior to admission.

### Follow-up

Post-interventional imaging was obtained within 24 h after endovascular therapy (EVT). Of the 49 patients, 29 showed no hemorrhage (59.2%). 15 patients (30.6%) experienced hemorrhagic transformation. Radiologically significant hemorrhage was detected in 5 cases (10.2%), including one fatal hemorrhage in a patient without eptifibatide (Tables 2 and 3).

**Table 2:** Comparison of characteristics of patients with favorable and unfavorable 90 day outcome. Numbers are individual patient counts and percent in brackets, Inter Quartile Range in square brackets

Outcome	mRS 90-day outcome 0–2 (favorable)	mRS 90-day outcome 3–6 (unfavorable)	*P*-value
Total	31	18	
Age (median [IQR])	61.00 [51.50, 66.50]	63.50 [55.50, 68.00]	0.707
Sex = Male (%)	21 (67.7)	14 (77.8)	0.854
mRS on admission 3–6 (%)	22 (71.0)	15 (83.3)	0.854
mRS at discharge 3–6 (%)	14 (45.2)	16 (88.9)	0.018
NIHSS on admission (median [IQR])	11.00 [5.50, 16.00]	14.50 [11.00, 17.50]	0.524
NIHSS at discharge (median [IQR])	1.00 [0.50, 6.00]	10.50 [8.75, 14.25]	0.001
NIHSS at 90-day (median [IQR])	1.00 [0.00, 2.00]	6.00 [5.00, 8.00]	0.0005
Pre-ASPECTS (median [IQR])	8.00 [7.00, 9.00]	7.00 [6.00, 8.00]	0.234
IVT rt-PA (Alteplase)	17 (54.8)	11 (61.1)	0.887
Eptifibatide administration	13 (41.9)	7 (38.9)	1
IAT stenting (%)	22 (71.0)	11 (61.1)	0.854
Dual layer of stents (%)			0.887
No	17 (54.8)	8 (44.4)	
Yes	5 (16.1)	3 (16.7)	
Stents number (%)			0.887
0	9 (29.0)	7 (38.9)	
1	14 (45.2)	5 (27.8)	
2	5 (16.1)	4 (22.2)	
3	2 (6.5)	2 (11.1)	
4	1 (3.2)	0 (0.0)	
TICI (%)			0.014
0	1 (3.2)	4 (22.2)	
1	1 (3.2)	0 (0.0)	
2b	3 (9.7)	8 (44.4)	
2c	4 (12.9)	1 (5.6)	
3	22 (71.0)	5 (27.8)	
Post-OP reocclusion within 24 h (%)	2 (6.5)	3 (16.7)	0.711
ICA occlusion posttreatment (%)	5 (16.1)	4 (22.2)	0.887
Recurrent stroke (%)	2 (6.5)	2 (11.1)	0.887
Bleeding Classification Heidelberger (%)			0.189
No bleeding (0)	22 (71.0)	7 (38.9)	
Hemorrhagic transformation (1)	8 (25.8)	7 (38.9)	
Radiologically significant bleeding (2/3)	1 (3.2)	4 (22.2)

**Table 3:** Comparison of characteristics of patients which did not receive eptifibatide (no) and which received eptifibatide (yes). Numbers are individual patient counts and percent in brackets, Inter Quartile Range in square brackets

Eptifibatide administration	No	Yes	*P*-value
Total	29	20	
mRS on admission 3–6 (%)	22 (75.9)	15 (75.0)	1
mRS at discharge 3–6 (%)	17 (58.6)	13 (65.0)	0.962
mRS at 90-day 3–6 (%)	11 (37.9)	7 (35.0)	1
NIHSS on admission (median [IQR])	11.00 [6.00, 16.00]	13.00 [5.75, 17.25]	0.962
NIHSS at discharge (median [IQR])	6.00 [1.00, 9.00]	4.00 [1.00, 11.00]	0.962
NIHSS at 90-day (median [IQR])	1.50 [0.00, 4.75]	2.50 [0.25, 4.75]	0.962
IAT stenting (%)			
Dual layer of stents (%)			0.097
No	16 (55.2)	9 (45.0)	
Yes	1 (3.4)	7 (35.0)	
Stents number (%)			0.097
0	12 (41.4)	4 (20.0)	
1	14 (48.3)	5 (25.0)	
2	2 (6.9)	7 (35.0)	
3	1 (3.4)	3 (15.0)	
4	0 (0.0)	1 (5.0)	
TICI (%)			0.962
0	4 (13.8)	1 (5.0)	
1	1 (3.4)	0 (0.0)	
2b	8 (27.6)	3 (15.0)	
2c	1 (3.4)	4 (20.0)	
3	15 (51.7)	12 (60.0)	
Post-OP reocclusion within 24 h (%)	2 (6.9)	3 (15.0)	0.962
ICA occlusion posttreatment (%)	5 (17.2)	4 (20.0)	1
Recurrent stroke (%)	3 (10.3)	1 (5.0)	0.962
Bleeding Classification Heidelberger (%)			
No bleeding (0)	16 (55.2)	13 (65.0)	
Hemorrhagic transformation (1)	10 (34.5)	5 (25.0)	
Radiologically significant bleeding (2/3)	3 (10.3)	2 (10.0)	

Nine patients experienced post-interventional ICA-occlusion (4 multi-stented occlusions, 2 with one stented occlusions, 3 patients without stent) within 24 h. Four of these patients received eptifibatide. Furthermore, four of these patients had an unfavorable outcome at discharge and at 90-day follow-up (22.2%). ICA occlusions with regard to stent number and types are summarized in Table 4.

**Table 4:** Presence of ICA occlusion on postinterventional imaging with regard to the number as well as the types of stents. Numbers are individual patient counts and percent in brackets

ACI occlusion post intervention	No	Yes	*P*-value
Number of stents (%)			1
0 (no stents)	13 (32.5)	3 (33.3)	
1	17 (42.5)	2 (22.2)	
2	6 (15.0)	3 (33.3)	
3	3 (7.5)	1 (11.1)	
4	1 (2.5)	0 (0.0)	
Dual layer (%)			0.89467
No	22 (55.0)	3 (33.3)	
Yes	5 (12.5)	3 (33.3)	

### Outcome measures

Thirty-one patients (63.3%) showed a favorable 90-day modified Rankin Scale (mRS 0–2) clinical outcome. Out of these 13 patients (41.9%) received eptifibatide periinterventionally. One patient (3.2%) showed radiologically significant hemorrhage after administration of eptifibatide (Table 2). In 29 of these patients, successful reperfusion (TICI-Score: 2b-3) was achieved.

Eighteen patients (36.7%) showed an unfavorable 90-day mRS (3–5) clinical outcome. Among these, seven (38.9%) received eptifibatide. Four patients (22.2%) had radiologically significant hemorrhage, one of whom received eptifibatide. In 14 of these patients, successful reperfusion (TICI 2b—3) was achieved.

Successful reperfusion was significantly higher (adjusted *p*-value = 0.014) in the favourable 90-day mRS group compared to the unfavorable 90-day mRS group (93.6 vs 77.8% TICI 2b-3). No statistical differences between the two groups in terms of Aspirin and Heparin use were detected (*p*-values of 0.87 and 1, respectively).

The median NIHSS scores at admission did not show a significant difference between patients with favorable and unfavorable modified Rankin Scale (mRS) clinical outcomes (median NIHSS at admission: 11 vs. 14.5).

## Discussion

This single-center cohort investigated the clinical and radiological outcomes following EVT of symptomatic cervical dissection with regard to eptifibatide administration. The main finding of our study is, that the use of a GP IIb/IIIa inhibitor (eptifibatide) during endovascular treatment of cervical arterial dissections does not result in a higher incidence of unfavorable clinical outcome or intracranial hemorrhage compared to those who did not receive a GP IIb/IIIa inhibitor.

In the present study, roughly 40% of patients received eptifibatide. The rate of patients receiving eptifibatide did not differ significantly between the patient groups with favorable and unfavorable clinical outcome. Furthermore, the rate of radiologically significant hemorrhage did not differ significantly between patients that received eptifibatide and those who did not.

In turn, the incidence of radiologically significant hemorrhage was higher in patients with an unfavorable 90-day mRS clinical outcome compared to those with a favorable 90-day mRS outcome, even though this did not reach statistical significance. Also, the study revealed that the majority of patients with an unfavorable mRS outcome at discharge still had an unfavorable mRS at 90 days.

**Fig. 2:**
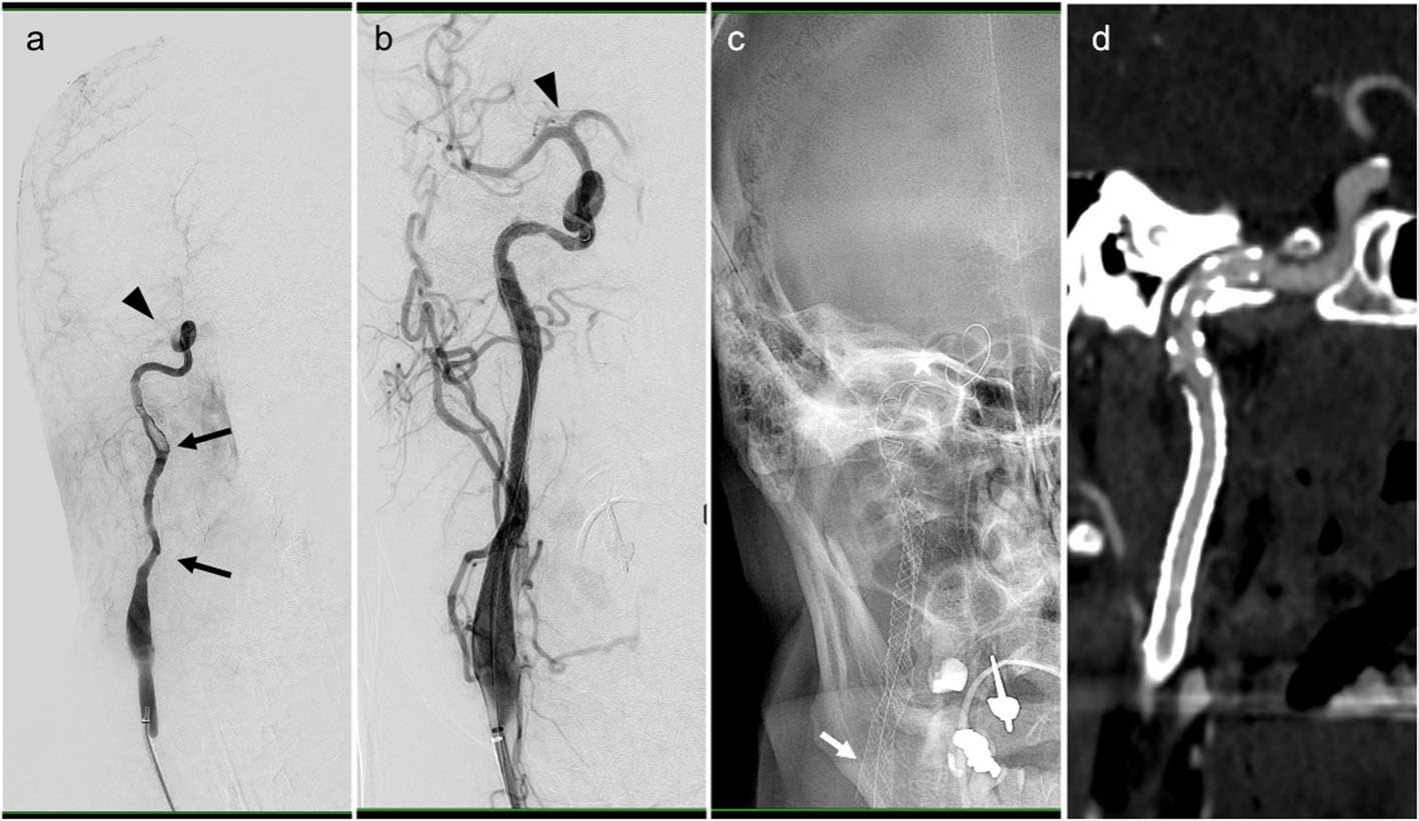
DSA images of a male patient with cervical ICA dissection (arrows in **a**) and carotid-T occlusion (arrowhead in **a**). The MCA was recanalized (arrowhead in **b**) and the ICA reconstructed using three overlapping stents (**c**: arrow shows proximal end, asterisk distal end). The patient was treated with i.v. Lysis and therefore received Aspirin only at the beginning of the intervention. Due to extensive clot formation, i.v. eptifibatide was started prior to stent placement. Control CTA 12 h post intervention showed patency of the stents (**d**). DSA: Digital subtraction angiography, ICA: Internal carotid artery, MCA: Middle cerebral artery, CTA: Computerized Tomography Angiography

In the study population, extracranial internal carotid artery with middle cerebral artery occlusion (tandem pathology) predominated (Fig. 2). This type of occlusion is likely seen in patients with CeAD stroke and is considered a challenging type of blockage to treat due to its location and complexity [34]. Different treatment strategies may be required to achieve successful recanalization.

In our study, successful recanalization (defined as achieving a TICI 2b-3 grade) was found to be significantly correlated with favorable 90-days outcomes. Specifically, 93.6% of patients with favorable 90-days outcome were successfully recanalized. The post-operative occlusions within 24 h were associated with an unfavorable clinical outcome. These results suggest that the effectiveness of recanalization and the maintenance of ICA patency are critical factors that appear to significantly influence the functional outcomes of patients undergoing EVT [35]. In this series, 16 patients were not treated with stenting of the dissection. Main criterium for not stenting the cervical dissection was sufficient antegrade flow as regarded by the interventionist.

**Fig. 3:**
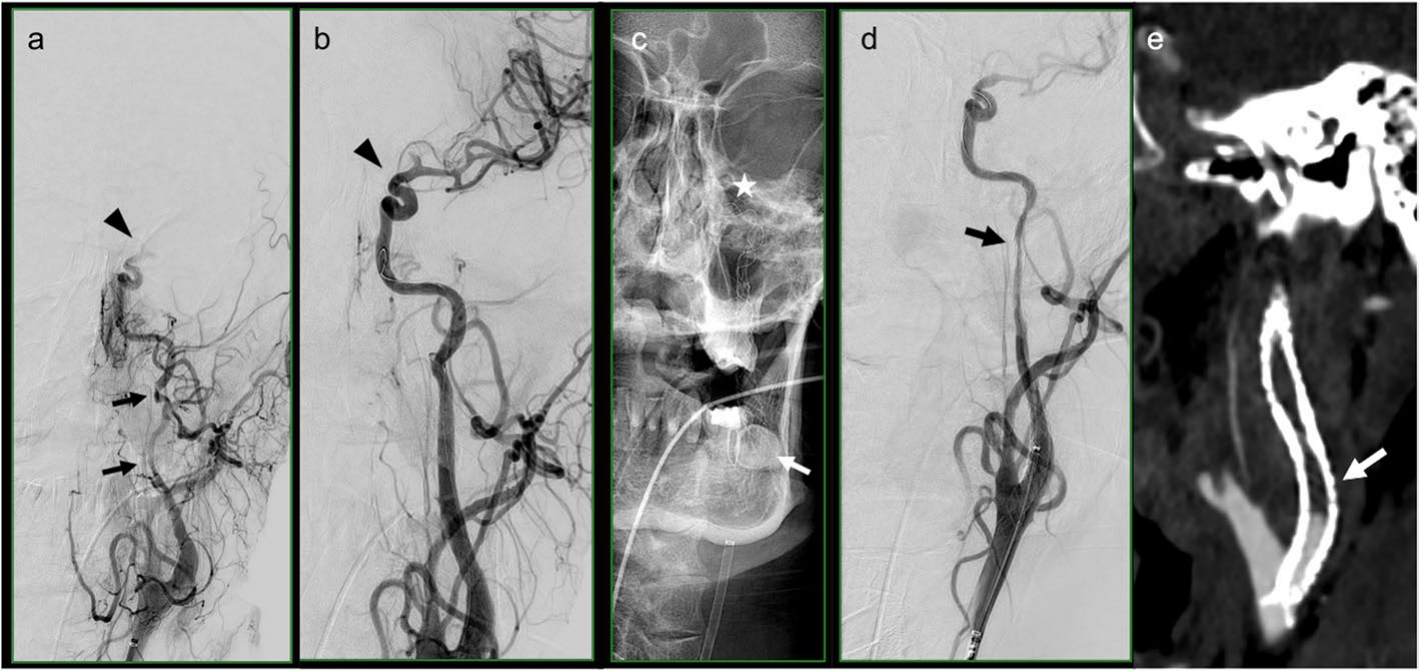
DSA image of a 56y old patient with cervical ICA dissection (arrows in **a**) and MCA occlusion (arrowhead in **a**). The MCA was recanalized (arrowhead in **b**) and the ICA reconstructed using three overlapping stents (**c**: arrow shows proximal end before placement of 3rd stent, asterisk distal end). Despite i.v. eptifibatide infusion and prior aspirin intake, in-stent thrombosis occurred at the end of the intervention (arrow in **d**). Control CTA 12 h post intervention showed occlusion of the stents (arrow in **e**). DSA: Digital subtraction angiography, ICA: Internal carotid artery, MCA: Middle cerebral artery, CTA: Computerized Tomography Angiography

In our study, the recommended eptifibatide dose regimen based on cardiology studies was administered [29, 36]. Nevertheless, we found three patients with stent occlusions under eptifibatide infusion (Fig. 3). Two of them were complex recanalizations requiring multiple stents along the cervical and petrous ICA. In light of this, our data may indicate that the likelihood of administering eptifibatide increases with the number of stents as well as the use of dual-layer stents. Furthermore, it highlights the importance of reaching a sufficiently high GP IIb/IIIa receptor blockage in this highly thrombogenic situation that may require complex stent reconstructions. Intraprocedural thrombocyte function testing might avoid under- or overdosing of GP IIb/IIIa inhibitors, however, these tests are at present frequently not available in emergency situations.

Eptifibatide was taken from the market in late 2023, leaving tirofiban as the only remaining GP IIb/IIIa inhibitor available. These two drugs, however, seem to be comparable regarding safety and efficacy and have been extensively studied in stroke [37–39]. Furthermore, it is possible that eptifibatide may be available at some point in the future by a different distributor.

In the present study, the use of other antithrombotic drugs (Heparin, Aspirin) was not found to be associated with neither worse clinical outcome nor intracranial hemorrhage. In contrast, Heparin was found to be associated with an increased rate of intracranial hemorrhage and worse clinical outcome in the prospective MR clean med trial [40]. However, this result may not be directly applicable to our cohort, which predominantly consists of patients with highly thrombogenic tandem lesions that typically require a more aggressive antithrombotic regimen. Close monitoring of these patients remains essential, and further studies are needed to better understand the relationship between these treatments and bleeding complications. The optimal antithrombotic protocol for acute stenting in stroke patients remains to be identified in future randomized controlled studies.

Stroke due to CeAD is typically caused by a thromboembolic event [41]. In contrast to arteriosclerotic stenosis or occlusion, cervical artery dissections are not associated with a long-standing hypoperfusion state that leads to adaption of the cerebral vasculature. Therefore, the risk of hyperperfusion injury after recanalization may be lower after endovascular therapy of cervical dissections as compared with arteriosclerotic lesions [42, 43]. Moreover, CeAD is a common stroke etiology among young adults, which may indicate better overall health and shorter recovery time compared to older individuals. These factors suggest a high potential of favorable functional outcome for CeAD patients.

Furthermore, it is important to recognize that the risk of post-interventional hemorrhage after EVT for CeAD is influenced by various factors. These factors include the severity and location of the dissection, clot size, as well as the patient’s overall health and medical history. Patients who are taking anticoagulant medications or have a bleeding disorder may face an increased risk of hemorrhagic complications [44].

Shortcomings of the present study are the retrospective observational and non-randomized cohort, which could lead to selection bias. The sample size was also relatively small, and the subgroups used for analysis were therefore limited in size. Moreover, the low incidence of hemorrhages in the groups that did and did not receive GP IIb/IIIa inhibitors impedes the detection of statistically significant differences.

To summarize, the use GP IIb/IIIa inhibitors during emergency treatment of cervical artery dissections did not lead to a higher incidence of intracranial hemorrhage or worse 90-day clinical outcome in our retrospective study. However, these results are based on a limited sample size in a monocentric study and need to be further evaluated in larger, prospective trials.

## Conclusion

This retrospective cohort study showed no significant difference in the occurrence of hemorrhage or unfavorable 90-day clinical outcomes when a GP IIb/IIIa inhibitor (eptifibatide) was used during EVT in CeAD-related strokes. However, effects may be detected on larger cohorts. Successful reperfusion was the sole parameter significantly associated with a favorable clinical outcome. Our study provides evidence, that GP IIb/IIIa inhibitors may be added to Aspirin during treatment of symptomatic cervical artery dissections that require stenting without significantly increasing the risk of intracranial hemorrhage. In the present study, a GP IIb/IIIa inhibitor was used if thrombotic phenomena appeared after Aspirin loading dose, indicating insufficient antiplatelet function. As a limitation, Eptifibatide is not available anymore at present. However, the remaining GP IIb/IIIa inhibitor Tirofiban shows a similar safety and efficacy profile in ischemic stroke.

## Data Availability

Not applicable.

## Abbreviations

EVT: Endovascular therapy
CeAD: Cervical artery dissection
GP: Glycoprotein
mRS: Modified Rankin Scale
NIHSS: National Institute of Health Stroke Scale
